# Integrative Multiomics Analysis Identifies DKK3 as a Germline Susceptibility‐Related Regulator of Risperidone Response and Metabolic Risk in Schizophrenia

**DOI:** 10.1155/humu/8434158

**Published:** 2026-06-17

**Authors:** Xiaoyun Zhang, Jingjing Li, Yao Sun, Gangrui Hei, Xueqin Song

**Affiliations:** ^1^ Department of Psychiatry, The First Affiliated Hospital of Zhengzhou University, Zhengzhou, China, zzu.edu.cn; ^2^ Biological Psychiatry International Joint Laboratory of Henan, Zhengzhou University, Zhengzhou, China, zzu.edu.cn; ^3^ Henan Psychiatric Transformation Research Key Laboratory, Zhengzhou University, Zhengzhou, China, zzu.edu.cn

**Keywords:** DKK3, germline mutation, metabolic dysregulation, risperidone, schizophrenia

## Abstract

**Background:**

Risperidone is a widely used second‐generation antipsychotic for schizophrenia, known for its clinical efficacy but also for long‐term metabolic side effects, including weight gain and glucose–lipid dysregulation. Although its therapeutic effects have been well characterized, the molecular basis linking its benefits and adverse metabolic outcomes remains poorly understood.

**Methods:**

To identify genes mediating both therapeutic and metabolic responses to risperidone, we conducted an integrative multiomics study combining large‐scale genetic association data with transcriptomic and epigenetic profiling. Differentially expressed genes following risperidone exposure were intersected with schizophrenia‐ and metabolism‐associated loci using summary data‐based Mendelian randomization analysis based on GWAS summary statistics and eQTL data to uncover germline variants, germline susceptibility, and potential germline alteration. DNA methylation profiling from patient‐derived peripheral blood mononuclear cells (PBMCs) was used for regulatory validation.

**Results:**

We identified 120 genes significantly modulated by risperidone, among which DKK3, EEF1A1, and PRKAA1 were causally associated with schizophrenia and metabolic traits through germline mutation‐related regulatory evidence. Notably, DKK3 was downregulated after risperidone exposure and exhibited promoter hypermethylation, consistent with epigenetic regulation interacting with germline alteration and germline susceptibility. Functional correlation analysis revealed that lower DKK3 expression was associated with glycolipid dysregulation, supporting its role as a molecular bridge between antipsychotic action and metabolic liability.

**Conclusion:**

Our findings identify DKK3 as a germline mutation‐related and epigenetically regulated candidate that bridges risperidone′s neuropsychiatric benefits with its metabolic risks. This work offers novel insight into the shared molecular basis of antipsychotic response and side effects and suggests DKK3 as a promising biomarker for personalized treatment strategies in schizophrenia informed by germline regulatory variation and potential germline alteration.

## 1. Introduction

Schizophrenia is a severe psychiatric disorder with a chronic course, characterized by psychotic manifestations, negative symptoms, and cognitive deficits that substantially interfere with social adaptation and occupational performance [1–3]. Nowadays, antipsychotic medications are widely recognized as the primary treatment modality for schizophrenia among all treatments, with risperidone emerging as a widely used second‐generation antipsychotic due to its efficacy and relatively favorable side‐effect profile [4]. It has been observed that risperidone could effectively alleviate positive symptoms [5, 6], moderately improve negative symptoms [7, 8].

Although risperidone is widely used in clinical practice, extended treatment may be accompanied by metabolic complications, particularly weight gain, impaired glucose regulation, and lipid metabolic abnormalities [9, 10]. These complications not only elevate risks for cardiovascular and endocrine diseases but also diminish treatment adherence and quality of life. Despite extensive clinical evidence regarding the therapeutic efficacy and metabolic risks associated with risperidone, the underlying molecular mechanisms that connect these effects remain poorly understood.

Emerging studies suggest that host germline variation may modulate both treatment response and susceptibility to side effects [11–13]. Specifically, genes involved in neurotransmission, immune signaling, and metabolic pathways may jointly influence individual responses to risperidone through germline mutation‐related regulatory mechanisms, including germline alteration, germline susceptibility, and germline regulation [11, 14]. Yet, few studies have systematically explored whether a shared set of molecular regulators underlies both its benefits and liabilities.

To address this gap, we performed an integrative analysis to identify risperidone‐responsive genes associated with schizophrenia risk and metabolic traits. We combined large‐scale genetic association data with drug‐induced transcriptomic profiling, and validated key regulatory changes using DNA methylation data from a risperidone‐treated patient cohort. Our analysis highlights DKK3 as a central regulatory node linking antipsychotic efficacy and glycolipid metabolism, offering new insight into biomarker‐guided precision treatment for schizophrenia.

## 2. Methods

### 2.1. Experimental Procedures

**Figure 1:**
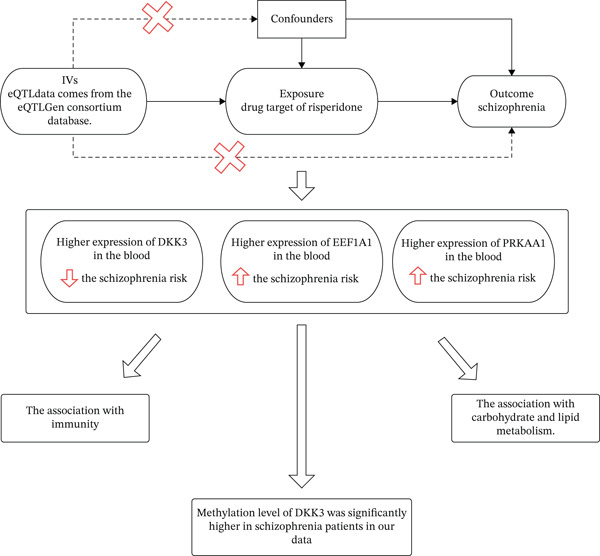
Study design and summary results.

We performed an integrative multiomics analysis to identify germline mutation‐related regulatory candidates that may link risperidone response, schizophrenia susceptibility, and metabolic risk. The analytical workflow consisted of several sequential steps. First, RNA‐seq data were used to identify risperidone‐responsive differentially expressed genes. Second, GWAS summary statistics and eQTL data were integrated through Mendelian randomization to evaluate whether candidate gene expression was supported by germline regulatory evidence associated with schizophrenia risk and germline mutation‐related susceptibility, including potential germline alteration. Third, pathway enrichment analyses were performed to explore the biological functions of the candidate genes. Fourth, glycolipid metabolism correlation analysis and CIBERSORT‐based immune infiltration analysis were used to evaluate their metabolic and immune relevance. Finally, transcription factor prediction, miRNA‐mRNA regulatory network analysis, and DNA methylation profiling were integrated to further characterize the regulatory features of the prioritized candidates. This stepwise workflow allowed us to prioritize DKK3 as a germline mutation‐related regulatory candidate supported by convergent transcriptomic, genetic, epigenetic, immune, and metabolic evidence. A detailed representation of the study design is provided in Figure 1.

### 2.2. Data Download

The eQTL data, sourced from the eQTLGen consortium (https://www.eqtlgen.org), serves as a primary resource for investigating the relationship between genetic variants and blood gene expression [15]. This dataset allows for a deeper understanding of how genetic variations influence gene activity, offering important insights into the genetic foundations of diverse traits and diseases. By leveraging this resource, we aim to uncover potential biomarkers and therapeutic targets associated with gene expression alterations in different biological contexts.

The outcome data is derived from GWAS summary datasets, which include more than 11 billion single nucleotide polymorphism (SNP)‐trait associations across 1673 GWAS studies. A significant portion of this dataset specifically focuses on schizophrenia (ebi‐a‐GCST90018919), encompassing 6334 cases and 445,120 controls.

Additionally, RNA‐seq data were obtained from the GEO database, which is managed by NCBI [16]. This dataset offers valuable gene expression profiles, enabling the investigation of transcriptomic variations across different conditions. We specifically downloaded the Series Matrix File for GSE19112, using GPL9692 as the annotation file. This dataset consists of gene expression profiles from 20 patients, comprising 10 from a control group and 10 treated with risperidone.

### 2.3. Analysis of Statistical Data

All statistical analyses were conducted using *R software* (Version 4.2.2). Differentially expressed genes were identified with the *Limma* package using a threshold of *p* < 0.05, and the results were visualized using *ggplot2* and *pheatmap*. Because of the limited sample size and exploratory nature of the RNA‐seq dataset, this nominal threshold was used for initial candidate screening. The resulting DEGs were not interpreted as definitive transcriptomic markers but were further prioritized through integrative multiomics evidence. Functional enrichment analyses, including GO and KEGG analyses, were performed using *Metascape,* with a minimum overlap of 3 and a significance cutoff of *p* ≤ 0.01. Mendelian randomization analysis was performed using summary statistics from the *IEU GWAS* database. Instrumental variables were selected at *p* < 1 × 10^−5^, followed by linkage disequilibrium clumping with an *R^2^* threshold of 0.001 and a 10,000 kb window. Causal effects were estimated using inverse‐variance weighted (IVW), MR‐Egger, and weighted median methods. Heterogeneity among instrumental variables was assessed using Cochran′s *Q* test.

*Gene set variation analysis (GSVA)* and *gene set enrichment analysis (GSEA)* were performed with gene sets sourced from the *Molecular Signatures Database (MSigDB)*. These analyses aimed to evaluate the overall activity of predefined gene sets across different conditions, providing insights into the molecular pathways and biological processes that are dysregulated in the studied samples. Correlation analyses of differentially expressed genes with metabolic pathways were performed using single‐sample *ssGSEA* and *GSVA*, employing datasets from *Reactome*. Immune cell infiltration analysis was carried out using the *CIBERSORT* algorithm. Transcription factor predictions were made using *RcisTarget*, and miRNA (MicroRNAs) regulatory networks were constructed based on data from the *miRcode* database. Differential methylation analysis was carried out using the *ChAMP* package, with visualizations created using *ggplot2*.

#### 2.3.1. Differential Expression Analysis and Functional Enrichment of Genes

The Limma package was utilized to analyze RNA‐seq data, aiming to identify genes with significant differential expression between the control and risperidone‐treated groups, applying a *p* value cutoff of < 0.05. This approach aimed to pinpoint genes whose expression levels are altered in response to risperidone treatment. To enhance the interpretability of the findings, the results were visualized through volcano plots and heatmaps, which were generated using the *Heatmap* package, providing clear representations of the most significantly upregulated and downregulated genes.

The differentially expressed genes were subsequently annotated for their biological functions using the Metascape database (https://www.metascape.org/) [17] which provides comprehensive functional annotation tools. GO and KEGG pathway analyses were carried out to identify key molecular functions, cellular components, and biological processes enriched in these genes, as well as key signaling pathways that may be involved in the observed gene expression changes.

#### 2.3.2. Mendelian Randomization Analysis

The MR analyses were based on three key assumptions: (1) Correlation assumption, the IV must exhibit a strong association with the exposure while maintaining no direct relationship with the outcome; (2) Independence assumption, the IV should be independent of any potential confounding variables that could affect both the exposure and the outcome; and (3) Exclusivity hypothesis, the IV′s effect on the outcome should be fully mediated through its influence on the exposure, with any deviation from this pathway indicating the potential for genetic pleiotropy. Throughout our analysis, we employed two‐sided statistical tests, applying a significance threshold of *p* < 0.05 for all assessments.

Mendelian randomization analysis was performed using summary‐level GWAS data from the IEU GWAS database. Outcome datasets were selected from available GWAS records for eQTL‐based causal inference. SNPs with *p* < 1 × 10^−5^ were selected as candidate instrumental variables, followed by linkage disequilibrium clumping using an *R* ^2^ threshold of 0.001 and a 10,000‐kb window. Causal estimates were obtained using IVW, MR‐Egger, weighted median, and weighted mode methods [18] . The Wald ratio method was applied when only one SNP was available for analysis. Heterogeneity was assessed using Cochran′s *Q* test [19]. This analysis was used to investigate whether cis‐ and trans‐regulated gene expression in peripheral blood may contribute causally to schizophrenia risk.

#### 2.3.3. GSVA and GSEA Pathway Enrichment Analysis

GSVA was used to evaluate sample‐wise pathway activity from transcriptomic data. Gene sets were downloaded from the *MSigDB*, and enrichment scores were calculated for each sample using the GSVA algorithm. Differential pathway activity between groups was subsequently assessed.

GSEA was applied to identify enriched pathways between high‐ and low‐expression groups of key genes. Genes were ranked by differential expression, and enrichment of predefined gene sets was tested using 1,000 phenotype‐based permutations.

#### 2.3.4. Analysis of the Relationship Between Differentially Expressed Genes and Glycolipid Metabolism

A correlation study was carried out to explore the connection between differentially expressed genes and glycolipid metabolism markers, focusing on their association with schizophrenia and metabolic processes. Target genes related to carbohydrate and lipid metabolism were obtained from REACTOME_METABOLISM_OF_CARBOHYDRATES and REACTOME_METABOLISM_OF_LIPIDS datasets. The ssGSEA and GSVA algorithms were used to calculate carbohydrate and lipid metabolism scores for each sample. Subsequently, the association between the expression levels of three key genes and carbohydrate and lipid metabolism was evaluated through correlation analysis.

#### 2.3.5. Analysis of Immune Cell Infiltration

CIBERSORT was applied to estimate the relative abundance of 22 immune cell subsets based on transcriptomic profiles. Pearson correlation analysis was then used to examine associations between immune cell fractions and gene expression levels.

#### 2.3.6. Investigation of the Regulatory Network Associated With Key Genes and miRNAs

##### 2.3.6.1. Exploration of the Regulatory Network Linked to Key Genes

The R package *RcisTarget* was used to identify candidate transcription factors [20] regulating key genes based on motif enrichment analysis. Motif enrichment was evaluated using the normalized enrichment score (NES), which was calculated from the AUC distribution of ranked motifs in the gene‐motif database. Motif annotation was further refined using motif similarity and gene sequence information.

##### 2.3.6.2. miRNA Network Construction

miRNAs are tiny RNA molecules without coding potential that play a key role in controlling gene expression. Their main roles involve facilitating the degradation of target mRNA or suppressing the translation of mRNA into proteins. To investigate the potential regulatory role of miRNAs on key genes, we retrieved relevant miRNAs from the miRcode database. A miRNA‐gene interaction network was subsequently constructed and visualized using Cytoscape software for further analysis [21].

#### 2.3.7. Differentially Methylated Genes (DMGs) Analysis

To investigate potential epigenetic mechanisms in schizophrenia, we conducted an analysis of DMGs. The ChAMP package was employed in this study to analyze a cohort of 145 schizophrenia patients and 143 healthy controls. We identified differentially methylated probes related to schizophrenia, applying a statistical significance cutoff of *p* − adjusted < 0.05. The methylation patterns for key genes were then visualized using the *ggplot2* package in R.

## 3. Results

Following the analytical workflow described in the Methods section, we first examined transcriptomic changes associated with risperidone treatment.

### 3.1. Analysis of Differential Gene Expression in Schizophrenia Patients Treated With Risperidone

**Figure 2:**
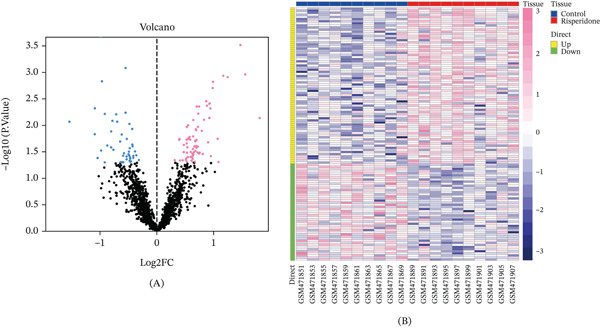
A) Volcano plot showing differentially expressed genes between the risperidone‐treated group and the untreated group. B) Heatmap showing the expression profiles of 120 differentially expressed genes, including 74 upregulated and 46 downregulated genes, across 20 schizophrenia patient samples. Rows represent genes, and columns represent individual samples. The top annotation bar indicates sample groups, and the side annotation bar indicates the direction of differential expression. Color intensity represents normalized relative gene expression levels.

We analyzed a schizophrenia‐related dataset obtained from the GEO public database, comprising 20 patients equally divided between a control group and a risperidone treatment group. Differential gene expression analysis was performed using the *Limma* package. Using a nominal significance threshold of *p* < 0.05, we identified 120 differentially expressed genes, including 74 upregulated and 46 downregulated genes based on the direction of log2 fold change. Given the relatively small sample size of the RNA‐seq dataset, these DEGs were interpreted as exploratory screening candidates rather than definitive transcriptomic signatures. Therefore, downstream candidate prioritization was based on convergent evidence across multiple analytical layers, including germline regulatory evidence, pathway enrichment, glycolipid metabolism correlation, immune infiltration, regulatory network analysis, and DNA methylation profiling. The volcano plot and heat map illustrate the distribution and expression patterns of these differentially expressed genes across samples (Figure 2A,B).

Functional enrichment analysis of these 120 genes using Metascape revealed enrichment in biological processes and pathways related to hormone response, focal adhesion, epithelial cell differentiation, T cell differentiation, response to nutrient levels, Wnt signaling, steroid hormone response, and phosphatidylinositol signaling (Figure S1). These enriched terms indicate that the differentially expressed genes were mainly associated with hormone‐related responses, cell adhesion, immune‐related differentiation, and metabolic regulation.

### 3.2. Identification and Predictive Performance Analysis of Key Genes for Schizophrenia

#### 3.2.1. Mendelian Randomization Analysis Identifies Key Genes Associated With Schizophrenia Risk

**Figure 3:**
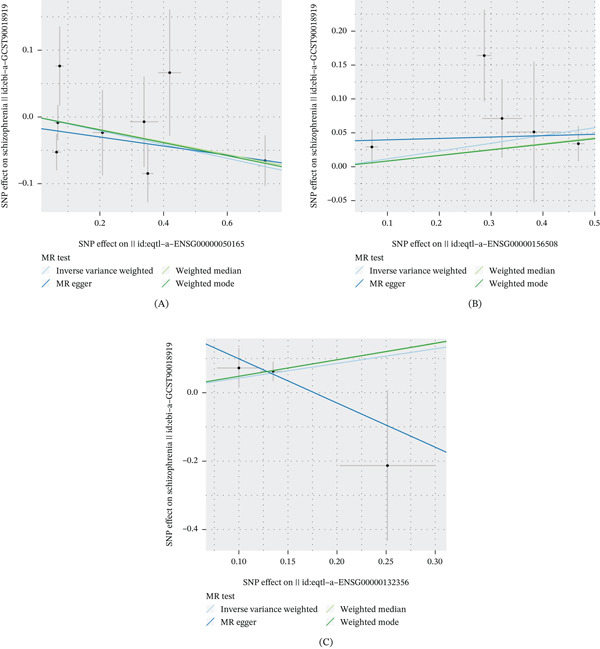
Mendelian randomization analysis of candidate genes associated with schizophrenia risk. MR scatter plots showing the associations of genetically predicted (A) DKK3, (B) EEF1A1, and (C) PRKAA1 expression with schizophrenia risk, respectively. Each point represents an instrumental variable SNP. The *x*‐axis shows the SNP effect on gene expression, and the *y*‐axis shows the SNP effect on schizophrenia risk. Lines represent causal estimates from IVW, MR‐Egger, weighted median, and weighted mode methods.

To elucidate the genetic factors influencing risperidone response in schizophrenia, we performed a Mendelian randomization analysis using summary statistics from 451,454 schizophrenia‐related samples (6334 cases and 445,120 controls; id: ebi‐a‐GCST90018919). This analysis identified three candidate genes associated with schizophrenia risk: DKK3, EEF1A1, and PRKAA1(Figure 3A–C). The results showed that DKK3 was associated with a reduced risk of schizophrenia (OR = 0.901, 95% CI: 0.822–0.989, *p* = 0.028; Figure 3A), whereas EEF1A1 and PRKAA1 correspond to an increased risk, with OR values of 1.122 (95% CI: 1.001–1.257, *p* = 0.047; Figure 3B) and 1.535 (95% CI: 1.015–2.321, *p* = 0.041; Figure 3C), respectively. The heterogeneity analysis indicated an absence of significant variability in these causal relationships, as shown in Figure S2A–F. Furthermore, a sensitivity analysis employing the leave‐one‐out method confirmed the reliability and stability of our results, with no single SNP substantially impacting the overall effect estimates (Figure S3A–C).

#### 3.2.2. Predictive Performance of Key Genes in Schizophrenia

We assessed the predictive ability of the three key genes by conducting ROC curve analysis to evaluate their performance. The results demonstrated good predictive capability for all three genes, with DKK3 showing an AUC of 0.760, whereas both EEF1A1 and PRKAA1 exhibited an AUC of 0.730 (Figure S4A–C). These results indicate that DKK3, EEF1A1, and PRKAA1 could act as potential biomarkers for schizophrenia risk and treatment response, with DKK3 showing slightly superior performance. The identification of these genes as potential predictive markers could have significant implications for personalized medicine approaches in schizophrenia treatment.

### 3.3. Pathway Enrichment and Functional Associations of Key Genes

#### 3.3.1. Enriched Pathways of DKK3, EEF1A1, and PRKAA1

**Figure 4:**
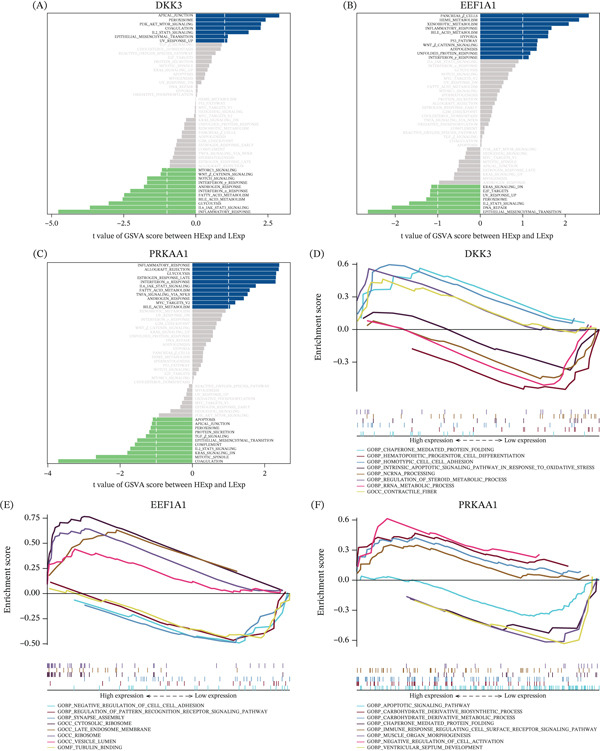
GSVA and GSEA pathway enrichment analyses of DKK3, EEF1A1, and PRKAA1. GSVA analysis showing pathway activity differences associated with (A) DKK3, (B) EEF1A1, and (C) PRKAA1, respectively. The bar plots display enriched pathways ranked according to the t value of GSVA scores. Blue bars indicate pathways positively associated with gene expression, whereas green bars indicate pathways negatively associated with gene expression. GSEA plots showing representative enriched pathways associated with (D) DKK3, (E) EEF1A1, and (F) PRKAA1, respectively. Enrichment curves illustrate the distribution of pathway‐related genes across the ranked gene list between high‐ and low‐expression groups.

GSVA and GSEA analyses revealed distinct functional enrichments for each of the three key genes, suggesting their involvement in schizophrenia‐related molecular processes. DKK3 was notably enriched in immune‐related pathways, such as IL2_STAT5_SIGNALING, in the GSVA analysis (Figure 4A), and also showed associations with NCRNA_PROCESSING, CHAPERONE_MEDIATED_PROTEIN_FOLDING, and RRNA_METABOLIC_PROCESS pathways in the GSEA analysis(Figure 4D), indicating potential roles in RNA regulation and protein homeostasis. EEF1A1 was linked to cell stress and signaling pathways, with enrichment in the P53_PATHWAY and WNT_BETA_CATENIN_SIGNALING in the GSVA analysis (Figure 4B), as well as ribosomal function, pattern recognition signaling, and vesicle transport processes identified through GSEA(Figure 4E). PRKAA1, a gene known for its metabolic regulatory roles, was associated with TNFA_SIGNALING_VIA_NFKB and BILE_ACID_METABOLISM in GSVA analysis (Figure 4C), and showed enrichment in pathways related to apoptosis, protein folding, and cardiac development in GSEA analysis (Figure 4F). These results collectively suggest that the three genes may contribute to schizophrenia pathophysiology through diverse yet biologically interconnected mechanisms involving immune regulation, metabolic control, and stress response, thereby offering potential targets for further functional exploration and therapeutic development.

#### 3.3.2. Association With Carbohydrate and Lipid Metabolism

**Figure 5:**
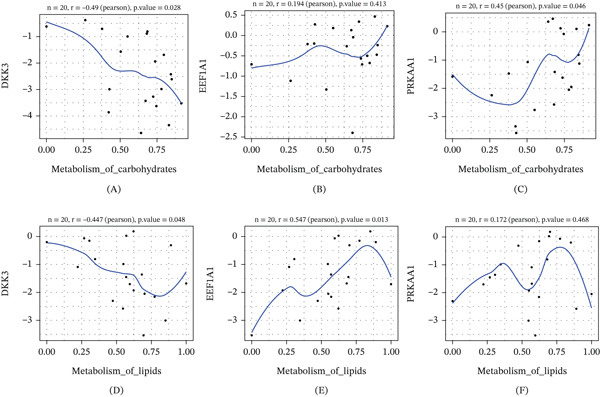
Correlations between candidate gene expression and metabolic pathway activity. Correlations between (A) DKK3, (B) EEF1A1, and (C) PRKAA1 expression and the carbohydrate metabolism pathway score, respectively. Correlations between (D) DKK3, (E) EEF1A1, and (F) PRKAA1 expression and the lipid metabolism pathway score, respectively. Each point represents an individual sample. Blue curves indicate fitted trend lines. Pearson correlation coefficients, sample size, and *p* values are shown in each panel.

ssGSEA revealed significant correlations between the key genes and metabolic processes. In terms of carbohydrate metabolism, DKK3 exhibited a notable negative correlation (*r* = −0.49, *p* = 0.028; Figure 5A), whereas PRKAA1 displayed markedly positive association (*r* = 0.45, *p* = 0.046; Figure 5C). No significant correlation was observed between EEF1A1 and carbohydrate metabolism (*r* = 0.194, *p* = 0.413; Figure 5B). For lipid metabolism, DKK3 exhibited a significant negative correlation (*r* = −0.447, *p* = 0.048; Figure 5D), whereas EEF1A1 showed a significant positive correlation (*r* = 0.547, *p* = 0.013; Figure 5E). No significant association was observed between PRKAA1 and lipid metabolism (*r* = 0.172, *p* = 0.468; Figure 5F). These findings suggest that DKK3, EEF1A1, and PRKAA1 may be differentially associated with carbohydrate and lipid metabolic processes, potentially contributing to metabolic alterations related to schizophrenia and antipsychotic treatment.

#### 3.3.3. Immune Cell Infiltration and Immune Regulatory Features

Analysis of immune cell infiltration revealed differences in plasma cells and resting mast cells between the risperidone‐treated and untreated groups (Figure S5A–C). These findings indicate that risperidone treatment may be associated with alterations in immune cell composition in schizophrenia patients. Further correlation analysis showed associations between the key genes and specific immune cell types. PRKAA1 was positively correlated with activated dendritic cells, suggesting its potential involvement in immune activation. In contrast, EEF1A1 showed negative correlations with resting dendritic cells and activated NK cells, indicating a possible association with immune cell composition changes. DKK3 was negatively correlated with neutrophils (Figure S5D). These results suggest that DKK3, EEF1A1, and PRKAA1 may be differentially associated with immune‐related alterations in schizophrenia.

Additional analysis using the TISIDB database showed associations between the key genes and immune‐related factors, including immune checkpoint‐related molecules and HLA‐related molecules (Figure S6A–B), further supporting a potential link between these genes and immune regulation.

### 3.4. Regulatory Network Analysis

**Figure 6:**
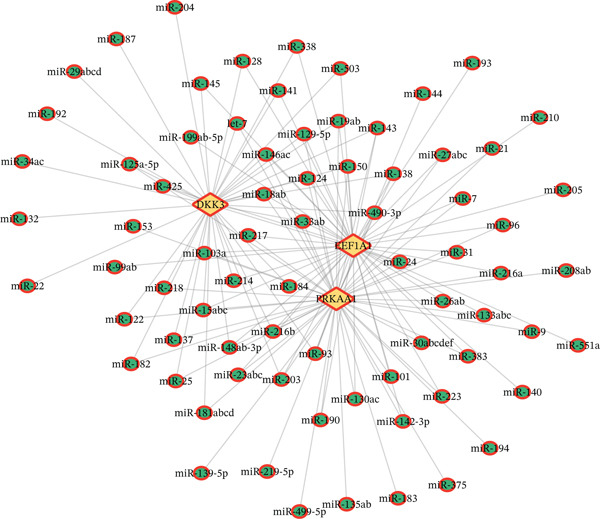
The mRNA‐miRNA relationship network of DKK3, EEF1A1, and PRKAA1 with 139 pairs.

Transcription factor (TF) enrichment analysis revealed that the motif cisbp__M6131 had the highest NES (NES = 5.77), suggesting a potential shared upstream regulatory element for DKK3, EEF1A1, and PRKAA1 (Supplementary Figure S7A–C). In parallel, miRNA–mRNA interaction analysis using the miRcode database identified a total of 139 interaction pairs involving 75 miRNAs targeting the three key genes (Figure 6), indicating a complex posttranscriptional regulatory landscape.

Notably, DKK3 appeared to be the most densely connected node within the network, suggesting extensive regulation by multiple miRNAs. These interactions imply that miRNAs may play both inhibitory and facilitative roles depending on cellular context, potentially modulating DKK3 expression in schizophrenia‐related immune and metabolic pathways. For instance, some miRNAs may act as silencers, whereas others may enhance expression under specific physiological or pathological conditions. This differential regulation could arise from factors such as binding affinity, miRNA abundance, and cell‐specific expression profiles. Together, these findings highlight a multilayered regulatory framework involving both transcriptional and posttranscriptional mechanisms, with DKK3 emerging as a central node with high regulatory complexity.

### 3.5. Differential Methylation Analysis

**Figure 7:**
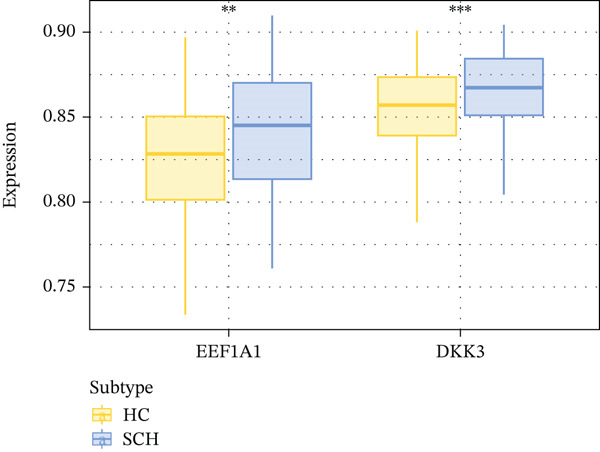
Analysis of differential methylation in DKK3, EEF1A1, and PRKAA1 between schizophrenia patients and healthy controls. ( ^∗^ *p* < 0.05,  ^∗∗^ *p* < 0.01,  ^∗∗∗^ *p* < 0.001).

Analysis of methylation data from 145 schizophrenia patients and 143 healthy controls revealed notable variations in the methylation levels of the three key genes: DKK3, EEF1A1, and PRKAA1. Notably, DKK3 and EEF1A1 showed significant methylation differences, with DKK3 being hypermethylated in schizophrenia patients, consistent with previous findings. However, EEF1A1 showed an opposite pattern, with hypomethylation observed in schizophrenia patients. PRKAA1 showed no significant variation (Figure 7).

These methylation patterns offer important insights into the epigenetic mechanisms governing gene expression in schizophrenia. The hypermethylation of DKK3 in schizophrenia patients suggests a potential transcriptional repression of this gene, which could lead to reduced expression. Given that DKK3 acts as a negative regulator in various biological processes, its reduced expression could consequently contribute to dysregulated immune response and metabolic processes associated with schizophrenia. Conversely, the hypomethylation of EEF1A1 in schizophrenia patients likely correlates with increased gene expression. EEF1A1 is essential for protein synthesis and regulating cellular metabolism; thus, its overexpression could pertain to altered cellular dynamics in schizophrenia, potentially affecting cell growth and function.

These differential methylation patterns not only corroborate our previous findings but also enhance our comprehension of the complex interplay between genetic and epigenetic factors in schizophrenia. The observed methylation changes in DKK3 and EEF1A1 may offer potential targets for therapeutic interventions aimed at normalizing gene expression levels. Further research could investigate the functional impacts of these methylation alterations and their feasibility as indicators for schizophrenia diagnosis or treatment response.

## 4. Discussion

This study employs MR analysis to explore the genetic mechanisms underlying risperidone treatment for schizophrenia, with a particular focus on the interaction between glycolipid metabolism and immune factors. Our analysis reveals that three key genes—DKK3, EEF1A1, and PRKAA1—are not only significantly associated with the risk of schizophrenia but also play crucial roles in glycolipid metabolism and immune regulation. These findings align with previous studies [22, 23] and offer new perspectives into the complex mechanisms of action of antipsychotic drugs, especially in terms of personalized treatment and minimizing side effects.

### 4.1. Genetic Associations and Their Implications

Our results indicate that DKK3 was associated with a reduced risk of schizophrenia, whereas EEF1A1 and PRKAA1 were associated with increased risk. Notably, DKK3 showed consistent signals across multiple analytical layers, supporting its potential protective association with schizophrenia. These genetic correlations provide important insights into the underlying mechanisms of the disorder, offering a deeper understanding of its pathophysiology and potential avenues for further research. Our analysis also revealed that DKK3 is significantly negatively correlated with glycolipid metabolism, providing a new direction for further investigation into DKK3′s mechanism of action. On the other hand, the positive correlation of PRKAA1 with glucose metabolism and EEF1A1 with lipid metabolism may explain the significant metabolic side effects experienced by some schizophrenia patients under risperidone treatment.

The methylation findings further support the regulatory relevance of DKK3 in schizophrenia. DKK3 hypermethylation in schizophrenia patients may represent an epigenetic regulatory layer that converges with germline mutation‐related susceptibility, germline alteration, and germline regulation. Although the current methylation dataset was not designed to determine whether this change was directly induced by risperidone, the observed methylation pattern is consistent with the broader multiomics evidence implicating DKK3 in disease vulnerability, treatment‐related molecular response, and metabolic risk. From this perspective, germline variants and potential germline alteration may shape the regulatory background of DKK3 expression, whereas DNA methylation may further modulate its transcriptional activity under disease‐ or treatment‐related conditions. Therefore, rather than being interpreted as an isolated methylation finding, DKK3 hypermethylation strengthens the view of DKK3 as a germline mutation‐related and epigenetically regulated candidate linking schizophrenia susceptibility, risperidone response, and immune‐metabolic dysregulation. The regulatory network analysis further supports the central regulatory relevance of DKK3. Rather than interpreting all predicted miRNA‐mRNA interactions equally, we focused on the DKK3‐centered regulatory relationships because DKK3 showed high network connectivity among the three candidate genes. miRNAs are important posttranscriptional regulators that can influence gene expression, immune responses, inflammatory signaling, and metabolic homeostasis. Therefore, DKK3‐targeting miRNAs may contribute to schizophrenia‐related immune‐metabolic dysregulation by modulating DKK3 expression. In this framework, germline variants and potential germline alteration may shape the regulatory background of DKK3, whereas miRNA‐mediated posttranscriptional regulation may further influence its expression under disease‐ or treatment‐related conditions. These findings suggest that the DKK3‐centered regulatory network may provide a mechanistic hypothesis linking germline susceptibility, epigenetic regulation, immune modulation, and metabolic vulnerability, although further experimental validation is required.

### 4.2. Metabolic Implications

Metabolic correlation analysis revealed important associations between key candidate genes and glycolipid metabolic pathways. DKK3 was negatively associated with both carbohydrate and lipid metabolism pathway scores, suggesting a potential link between DKK3 expression and glycolipid metabolic vulnerability during risperidone treatment. Importantly, this finding should be interpreted as associative rather than definitive causal evidence. Nevertheless, existing studies provide biological support for a possible metabolic role of DKK3. As a member of the Dickkopf family, DKK3 is closely related to Wnt/*β*‐catenin signaling, a pathway involved not only in neurodevelopment and synaptic regulation but also in adipogenesis, insulin sensitivity, lipid handling, and systemic metabolic homeostasis. Therefore, altered DKK3 regulation may provide a biological link between schizophrenia‐related molecular vulnerability and glycolipid metabolic disturbance during risperidone treatment.

Importantly, the positive correlation of PRKAA1 with glucose metabolism is consistent with previous evidence linking AMPK‐related genes to antipsychotic‐induced weight gain [23]. PRKAA1 encodes a catalytic subunit of AMPK, a central regulator of cellular energy balance, glucose utilization, and lipid metabolism. In addition, the association between EEF1A1 and lipid metabolism suggests that EEF1A1 may also be involved in metabolic alterations during risperidone treatment, although this finding requires further validation. Together, these findings suggest that DKK3, PRKAA1, and EEF1A1 may be differentially associated with glycolipid metabolic processes in schizophrenia patients receiving risperidone.

In this context, the association between lower DKK3 expression and glycolipid metabolism may reflect a regulatory pathway linking germline mutation‐related susceptibility, germline alteration, epigenetic regulation, and metabolic vulnerability. Moreover, the combined involvement of DKK3‐related Wnt/*β*‐catenin signaling, PRKAA1/AMPK‐associated energy regulation, and inflammatory pathways may provide a broader biological framework for understanding immune‐metabolic dysregulation during risperidone treatment. Future studies should use longitudinal clinical cohorts to determine whether DKK3 expression or methylation changes precede metabolic abnormalities during antipsychotic treatment. In addition, cellular and animal models could be used to manipulate DKK3 expression and examine its effects on Wnt/*β*‐catenin signaling, insulin sensitivity, lipid accumulation, inflammatory responses, and antipsychotic‐associated metabolic phenotypes.

### 4.3. Immune System Interactions

Our study also reveals important connections between these key genes and immune system function. Specifically, PRKAA1 showed a positive correlation with activated dendritic cells, whereas EEF1A1 was inversely associated with the activity of several immune cell types. In addition, CIBERSORT analysis showed differences in plasma cells and resting mast cells between the risperidone‐treated and untreated groups. Plasma cells are major effector cells of humoral immunity, and their alteration may reflect changes in adaptive immune activity during antipsychotic treatment. Mast cells are involved in inflammatory signaling, vascular permeability, and neuroimmune communication, and changes in resting mast cells may indicate altered inflammatory tone or immune‐metabolic balance in patients receiving risperidone.

These findings are consistent with previous evidence suggesting that immune dysfunction contributes to schizophrenia susceptibility and treatment‐related biological heterogeneity [24–26]. The interaction between glycolipid metabolism and immune factors further strengthens this complex interrelationship. Metabolic abnormalities may affect immune cell activity through inflammatory pathways, whereas immune activation and cytokine‐related changes may disrupt metabolic homeostasis, contributing to insulin resistance, dyslipidemia, or other metabolic disturbances [27, 28]. Since antipsychotic‐associated metabolic dysregulation is often accompanied by low‐grade inflammation, changes in plasma cells and mast cells may represent peripheral immune signatures associated with risperidone response and glycolipid metabolic vulnerability.

In this context, DKK3 may be particularly relevant because it emerged as a germline mutation‐related regulatory candidate associated with both immune‐related features and glycolipid metabolism. These immune infiltration findings should not be interpreted as direct mechanistic proof, but they support the possibility that germline susceptibility, immune regulation, and metabolic dysregulation may jointly contribute to heterogeneous responses to risperidone. The intricate relationship between metabolic disturbances and immune system dysfunction deepens our understanding of the pathological mechanisms of schizophrenia and suggests new avenues for exploring interventions that address both metabolic and immune‐related aspects of the condition [29].

### 4.4. Novel Treatment Perspective

Our findings propose a novel perspective for schizophrenia treatment by integrating germline susceptibility, metabolic status, and immune‐related features when evaluating risperidone response and side‐effect risk. Rather than serving as a standalone biomarker, DKK3 may be more appropriately considered as part of a multimarker panel that includes germline mutation‐related susceptibility, germline alteration, DNA methylation status, glycolipid metabolic indicators, immune signatures, and clinical treatment‐response profiles.

Such a panel could help stratify patients according to their vulnerability to risperidone‐associated metabolic side effects and support individualized monitoring during antipsychotic treatment. For example, patients with unfavorable DKK3‐related regulatory profiles may require closer follow‐up of body weight, glucose metabolism, lipid metabolism, and inflammatory status after risperidone initiation. This strategy may help clinicians optimize therapeutic benefit while reducing metabolic burden.

However, therapeutic targeting of DKK3 remains premature at the current stage. Further validation in longitudinal clinical cohorts and experimental models is required before DKK3 can be considered a direct intervention target. Overall, these findings support the potential value of incorporating germline regulation and immune‐metabolic profiles into precision treatment strategies for schizophrenia.

### 4.5. Study Limitations

Although this study provides valuable insights, several limitations should be acknowledged. First, the RNA‐seq dataset used for differential expression analysis was relatively small, including only 20 samples from the risperidone‐treated and control groups. This limited sample size may affect the robustness of DEG detection and increase the possibility of false‐positive findings, particularly because differentially expressed genes were identified using a nominal *p* < 0.05 threshold without multiple‐testing correction. Therefore, the DEG results should be interpreted as exploratory screening candidates rather than definitive transcriptomic signatures. To reduce overinterpretation, candidate genes were prioritized based on convergent evidence from transcriptomic alteration, germline regulatory evidence, pathway enrichment, glycolipid metabolism correlation, immune infiltration, regulatory network analysis, and DNA methylation profiling.

Second, although Mendelian randomization analysis provided supportive germline regulatory evidence, the possibility of horizontal pleiotropy cannot be completely excluded. Therefore, the MR findings should be interpreted cautiously as suggestive evidence for germline mutation‐related susceptibility and potential germline alteration rather than definitive causal proof. Future studies using larger GWAS datasets, more robust instrumental variables, and additional pleiotropy assessments such as MR‐PRESSO are needed to further validate these associations.

Third, although the methylation analysis was not based on a longitudinal risperidone exposure cohort, the observed DKK3 hypermethylation still provides important epigenetic evidence supporting the regulatory relevance of DKK3 in schizophrenia. This methylation pattern may reflect disease‐related epigenetic dysregulation that converges with germline susceptibility, germline mutation‐related regulatory background, potential germline alteration, medication exposure, immune status, and metabolic vulnerability. Future longitudinal methylation studies are needed to clarify the relative contributions of disease‐related and treatment‐related effects.

Finally, most public GWAS summary datasets and eQTLGen data used in this study primarily include individuals of European ancestry, which may limit the generalizability of the findings to other populations. Further validation in larger, ethnically diverse cohorts is required.

### 4.6. Future Directions

Future studies should focus on developing personalized treatment approaches based on identified genetic variations associated with risperidone′s therapeutic effects [30], future treatment can evolve towards more personalized approaches [31], potentially improving outcomes for patients with schizophrenia and reducing the risk of side effects.

Moreover, future studies research could provide deeper insights into the long‐term impact of medication treatment by conducting longitudinal follow‐up research. This is particularly relevant for chronic conditions such as schizophrenia, helping researchers monitor the sustainability of drug efficacy and its potential side effects, providing crucial information for improving patient quality of life and treatment adherence [32].

To address current limitations, future research should employ larger‐scale, multicenter study designs with diverse ethnic backgrounds. This approach would yield more representative data, enhancing the statistical power and universality of results.

Lastly, integrating genomic information with comprehensive clinical data could offer a more precise model for personalized schizophrenia treatment. This integration would allow for a deeper exploration of how genetic variations affect drug metabolic pathways and immune regulation.

## 5. Conclusion

This study employed Mendelian randomization and integrative multiomics analysis to identify germline mutation‐related regulatory candidates associated with risperidone response, schizophrenia susceptibility, and metabolic risk. Our findings revealed that DKK3, EEF1A1, and PRKAA1 were associated with schizophrenia risk and showed distinct relationships with glycolipid metabolism and immune regulation in the context of risperidone treatment.

Among these candidates, DKK3 showed relatively consistent evidence across transcriptomic, germline regulatory, epigenetic, metabolic, and immune‐related analyses. These findings support DKK3 as a germline mutation‐related and epigenetically regulated candidate that may link schizophrenia susceptibility, risperidone response, and metabolic vulnerability. In contrast, EEF1A1 and PRKAA1 may represent additional regulatory candidates involved in treatment‐related metabolic and immune alterations.

Overall, this study provides a multilayered molecular framework for understanding individual differences in risperidone response and metabolic side effects. The integration of germline susceptibility, potential germline alteration, DNA methylation, immune signatures, and glycolipid metabolic profiles may help guide future biomarker development and patient stratification strategies. Further validation in larger, longitudinal, and ethnically diverse cohorts, as well as functional experimental models, is required before these findings can be translated into clinical practice.

## Author Contributions

Xiaoyun Zhang designed the study, performed the data analysis, and drafted the manuscript. Jingjing Li, Yao Sun, and Gangrui Hei contributed to manuscript review and revision. Xueqin Song supervised the study, obtained funding, and critically reviewed and revised the manuscript. All authors read and approved the final manuscript.

## Funding

This work was supported by the National Natural Science Foundation of China, U21A20367 82471525 and the Scientific Research and Innovation Team of The First Affiliated Hospital of Zhengzhou University, ZYCXTD2023015.

## Disclosure

All authors approved the final version of the manuscript.

## Conflicts of Interest

The authors declare no conflicts of interest.

## Supporting information


**Supporting Information** Additional supporting information can be found online in the Supporting Information section. Figure S1 presents the enrichment analysis results of 120 candidate genes. Figures S2 and S3 display heterogeneity and sensitivity analyses for DKK3, EEF1A1, and PRKAA1 using funnel plots, forest plots, and leave‐one‐out methods. Figure S4 shows ROC curve analyses evaluating the predictive performance of these three genes. Figures S5 and S6 illustrate immune infiltration patterns and the associations between the key genes and immune‐related factors including immune checkpoints and HLA molecules. Figure S7 presents transcription factor enrichment analyses, including motif recovery curves, regulatory networks, and enrichment score summaries.

## Data Availability

The data that support the findings of this study are available from the corresponding author upon reasonable request.
