# Supplementary material for: Integrative Multiomics Analysis Identifies DKK3 as a Germline Susceptibility‐Related Regulator of Risperidone Response and Metabolic Risk in Schizophrenia
Source: Hum Mutat. 2026 Jun 17;2026:8434158. doi: 10.1155/humu/8434158 (PMC13273393; doi:10.1155/humu/8434158)
Supplement: Supplementary file 1 — Supporting Information Additional supporting information can be found online in the Supporting Information section. Figure S1 presents the enrichment analysis results of 120 candidate genes. Figures S2 and S3 display heterogeneity and sensitivity analyses for DKK3, EEF1A1, and PRKAA1 using funnel plots, forest plots, and leave‐one‐out methods. Figure S4 shows ROC curve analyses evaluating the predictive performance of these three genes. Figures S5 and S6 illustrate immune infiltration patterns and the associations between the key genes and immune‐related factors including immune checkpoints and HLA molecules. Figure S7 presents transcription factor enrichment analyses, including motif recovery curves, regulatory networks, and enrichment score summaries. [file HUMU-2026-8434158-s001.docx]

Supplementary Figure 1. Enrichment analysis of 120 genes.

A) Bar plot showing the top enriched GO terms and KEGG pathways ranked by −log10(P value). B) Enrichment network illustrating the relationships among enriched biological terms and pathways. Nodes represent enriched terms, and edges indicate functional similarities or shared genes between terms.


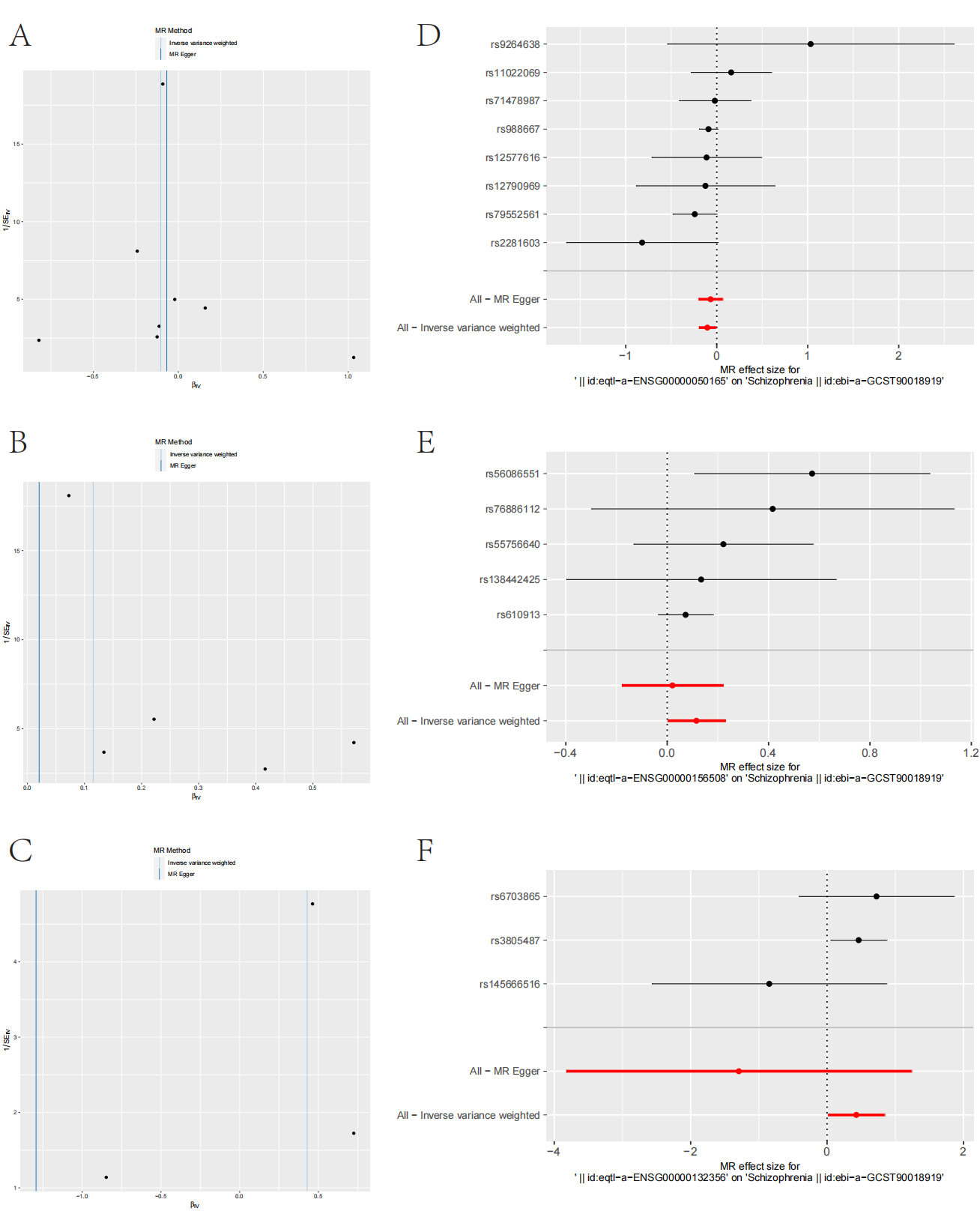


Supplementary Figure 2. Heterogeneity analysis of the genes DKK3, EEF1A1, and PRKAA1.

A-C) Funnel plots showing the distribution of individual SNP-level causal estimates for DKK3, EEF1A1, and PRKAA1, respectively. D-F) Forest plots showing the causal effect estimates of individual instrumental variable SNPs and the overall MR estimates for DKK3, EEF1A1, and PRKAA1, respectively. Black points represent SNP-specific estimates, and red points indicate the overall estimates derived from MR-Egger and inverse-variance weighted methods. Error bars represent 95% confidence intervals.


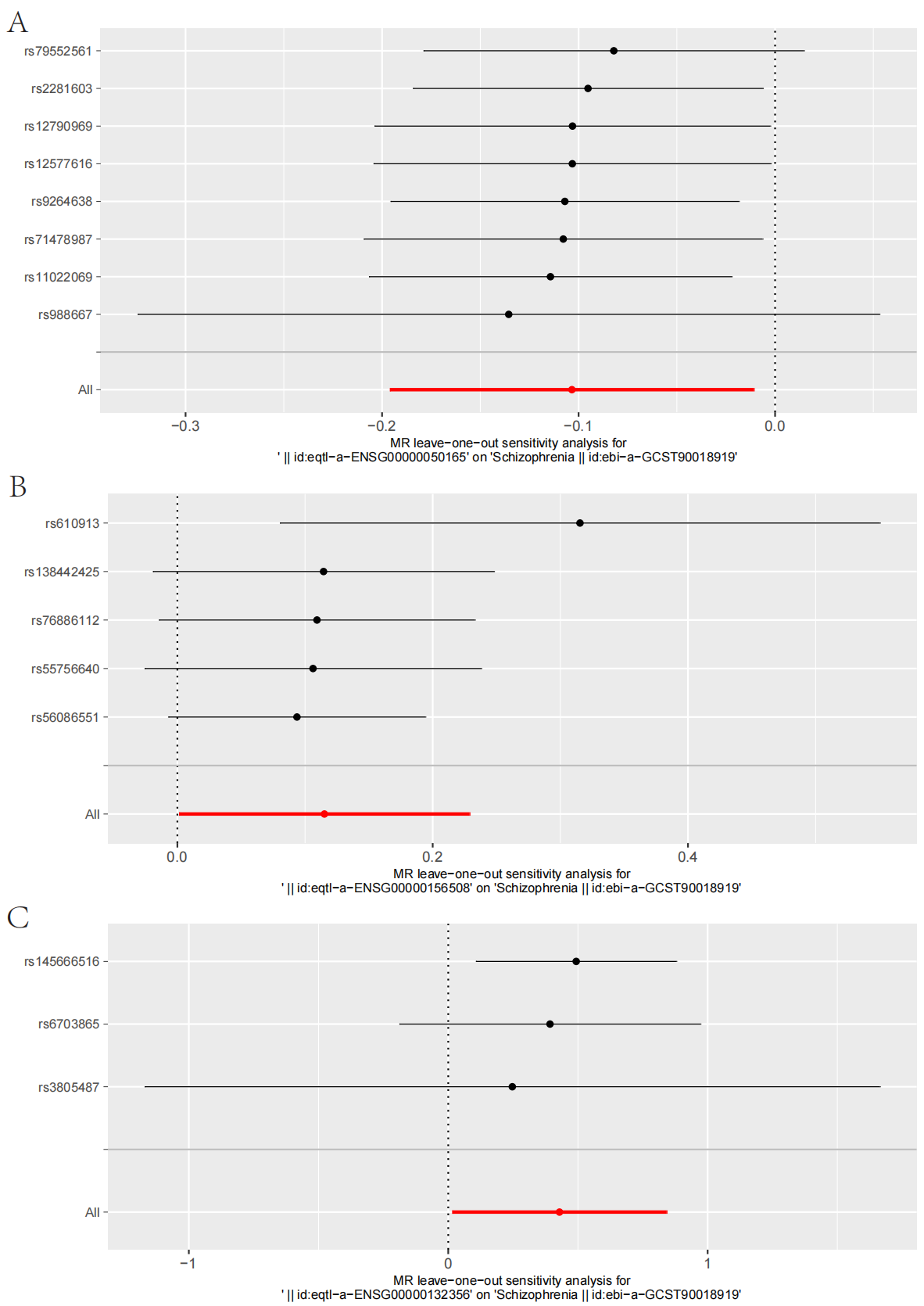


Supplementary Figure 3. Sensitivity analysis of the genes DKK3, EEF1A1, and PRKAA1 was performed using the leave-one-out method. A–C) Leave-one-out plots for DKK3, EEF1A1, and PRKAA1, respectively. Each black point represents the MR estimate after excluding one instrumental variable SNP. The red point represents the overall MR estimate using all instrumental variables. Horizontal lines indicate 95% confidence intervals.


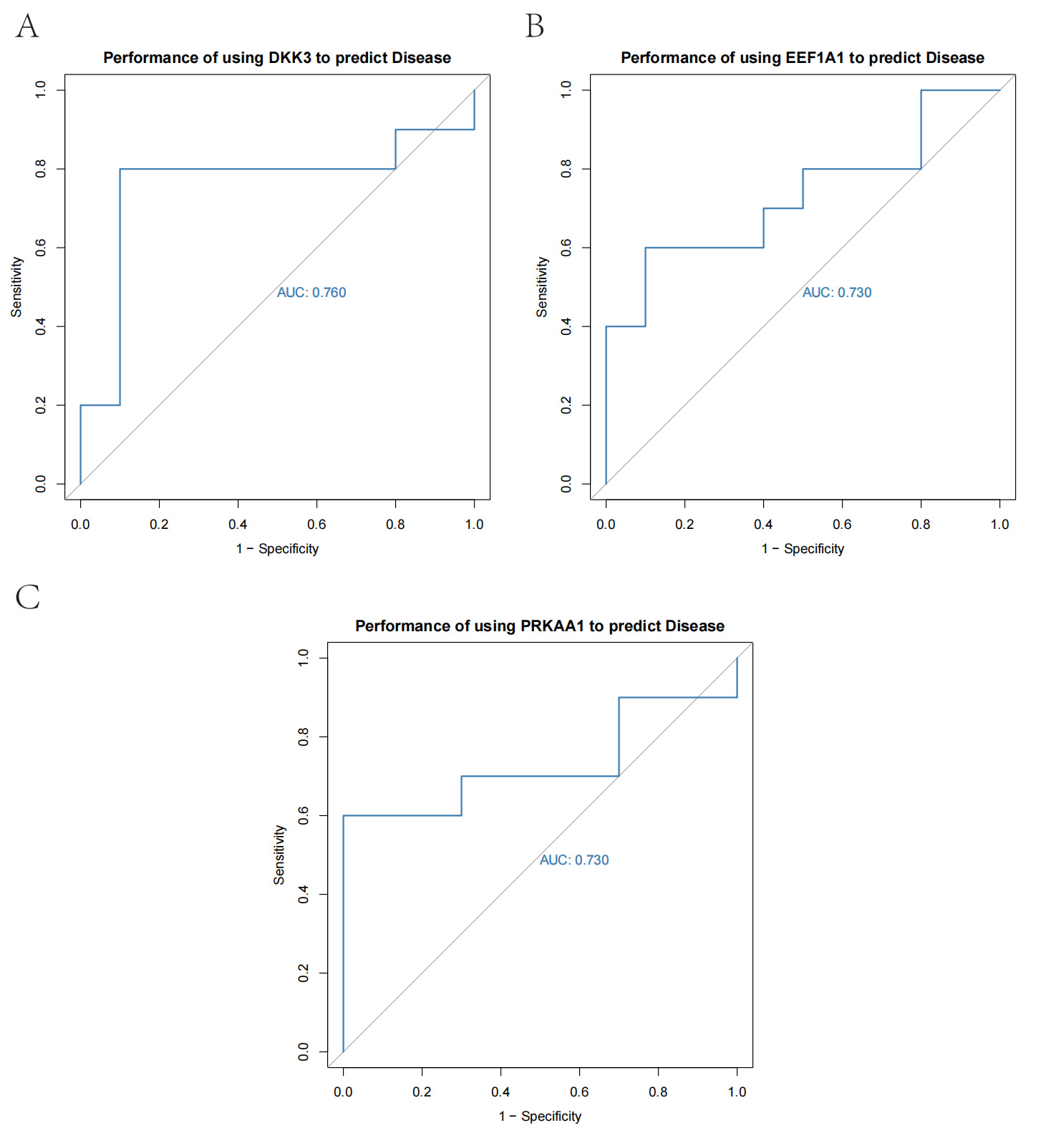


Supplementary Figure 4. ROC curve analysis was conducted to evaluate the predictive ability of DKK3, EEF1A1, and PRKAA1. ROC curves showing the predictive performance of DKK3, EEF1A1, and PRKAA1, respectively. The x-axis represents 1 − specificity, and the y-axis represents sensitivity.

| A  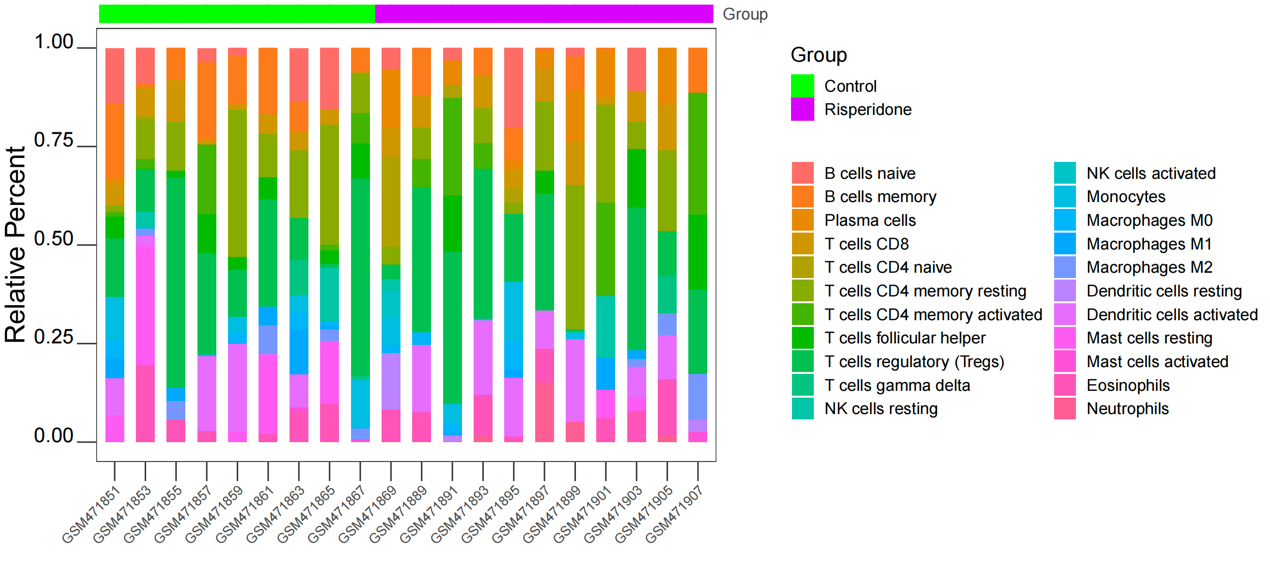 |
| --- |
| B  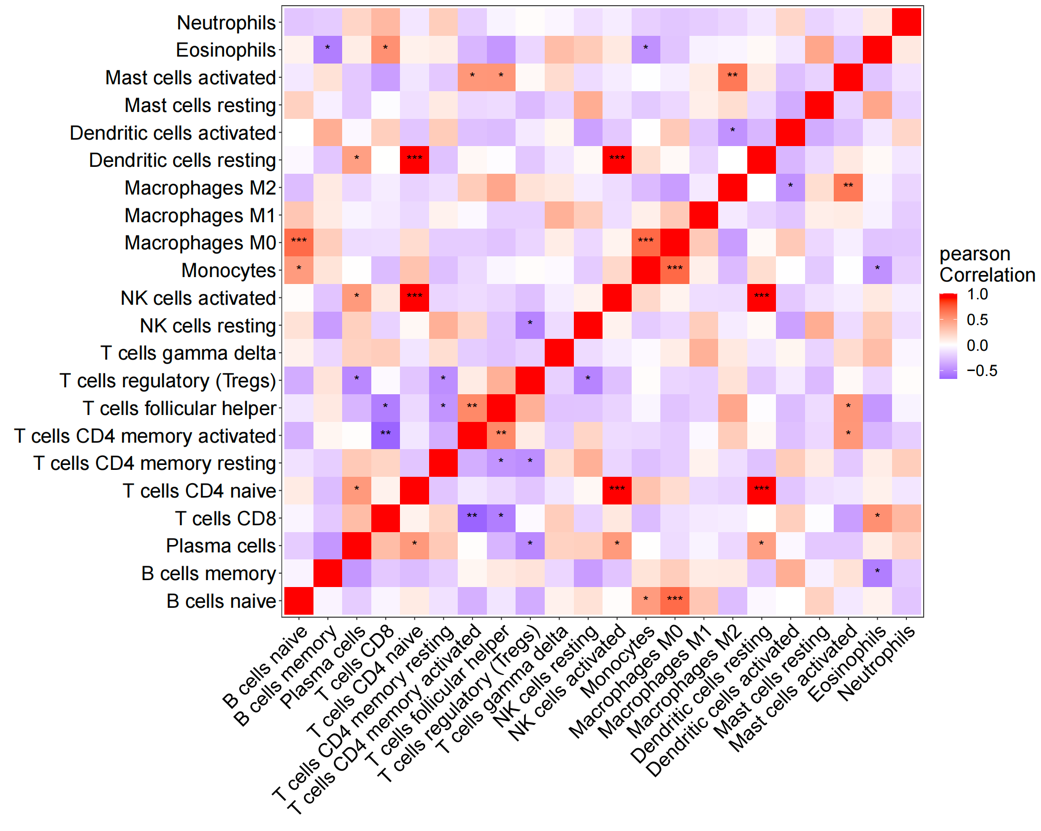 |
| C  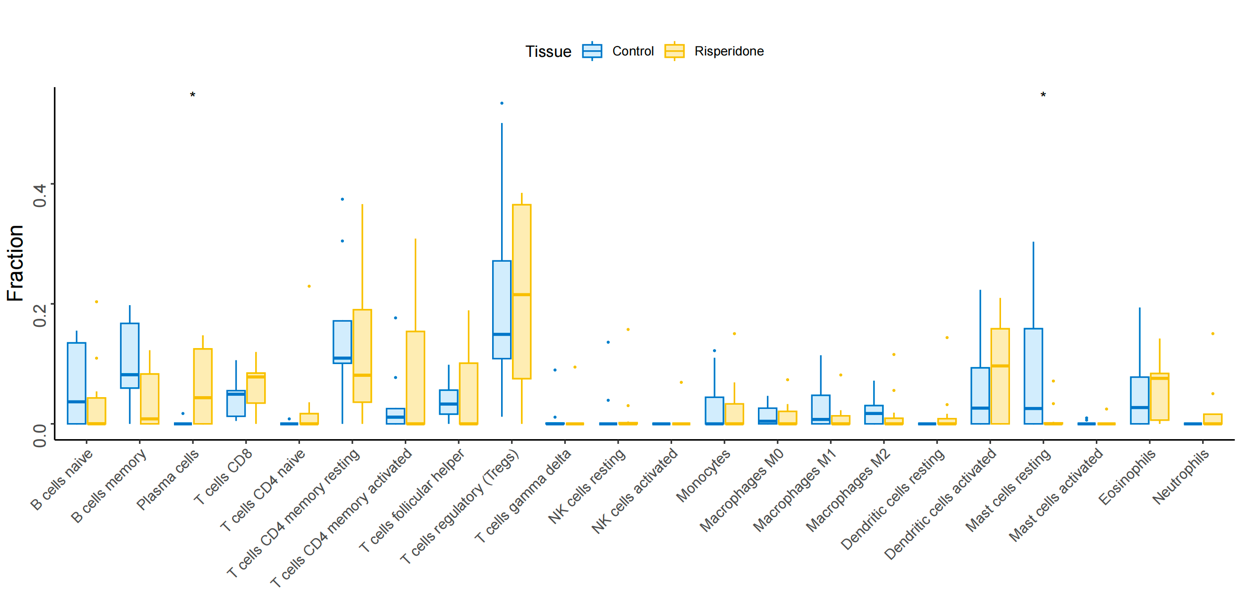 |
| D  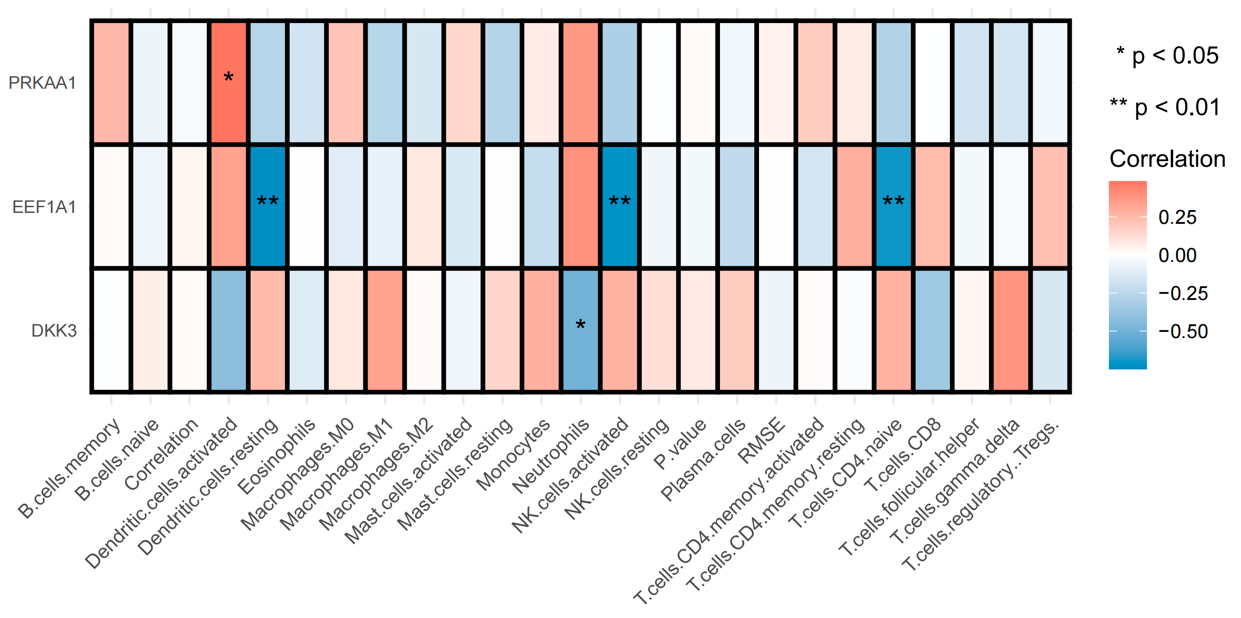 |

Supplementary Figure 5. The distribution of immune infiltration levels, the correlation between immune cells, and the correlation of DKK3, EEF1A1, and PRKAA1 with immune cells in risperdone group and control group. A) Stacked bar plot showing the relative proportions of 22 immune cell types in each sample. B) Correlation heatmap showing pairwise correlations among infiltrating immune cell types. C) Box plots comparing the relative fractions of immune cell types between the untreated and risperidone-treated groups. D) Correlation heatmap showing correlations between DKK3, EEF1A1, PRKAA1 expression and immune cell fractions. Asterisks indicate statistical significance.

| A  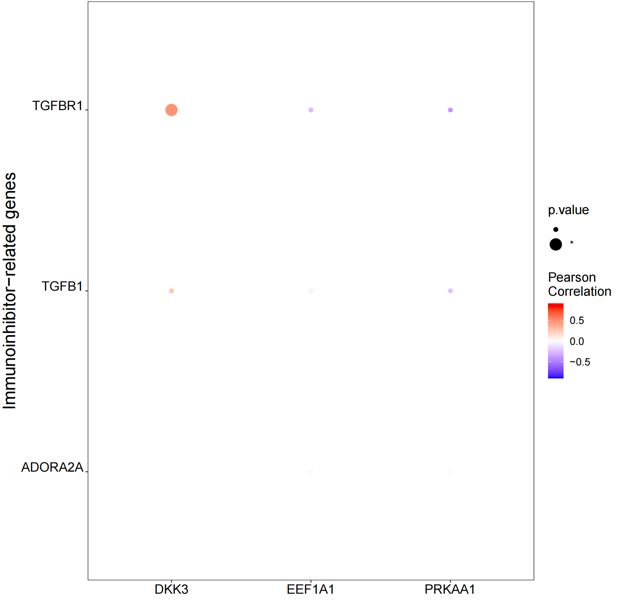 | B  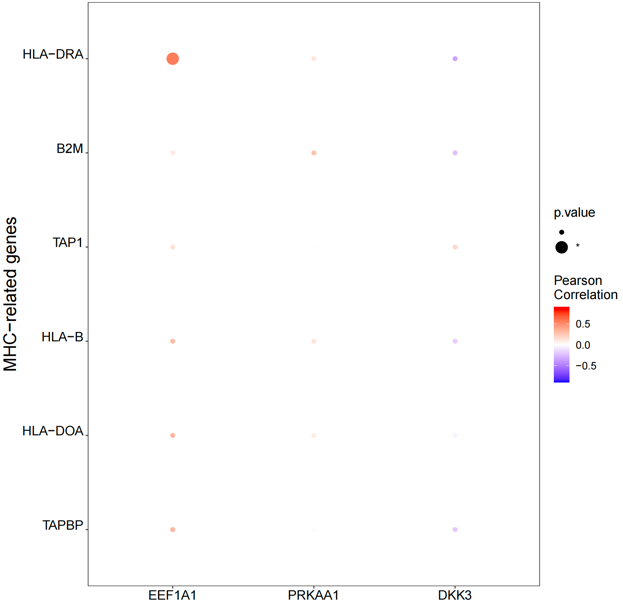 |
| --- | --- |

Supplementary Figure 6. The association between DKK3, EEF1A1, and PRKAA1 and immune-related factors. A) Bubble plot showing the associations between DKK3, EEF1A1, PRKAA1 and immune checkpoint-related molecules. B) Bubble plot showing the associations between DKK3, EEF1A1, PRKAA1 and HLA-related molecules. Bubble size represents the significance level, and color indicates the correlation coefficient.


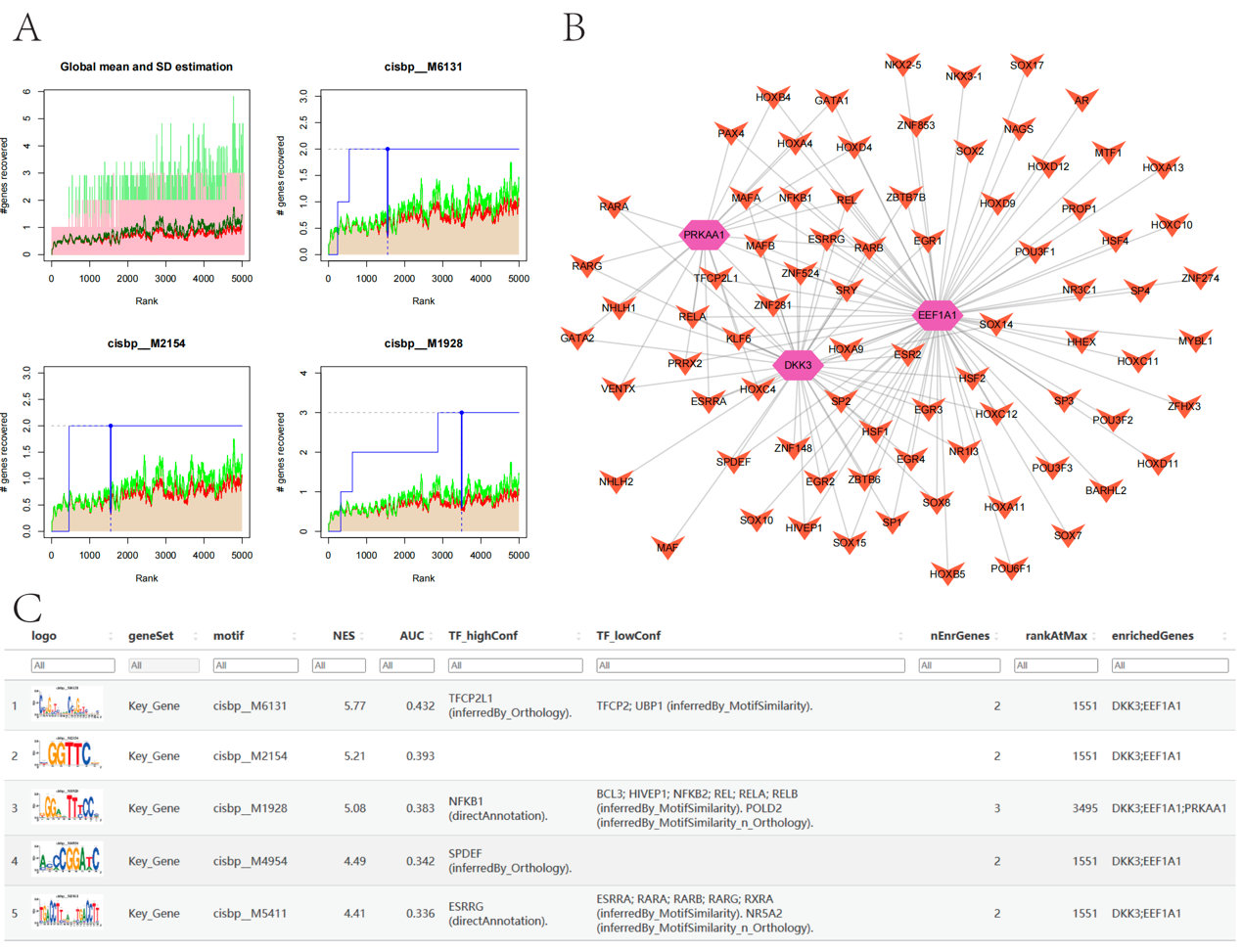


Supplementary Figure 7. Enrichment analysis of transcription factor. A) Recovery curves showing motif enrichment for candidate transcription factors identified by RcisTarget. B) Regulatory network showing predicted transcription factors and their target genes. C) Summary table of enriched transcription factor motifs, including normalized enrichment scores and associated annotations.
